# Escalating climate-related health risks for Hajj pilgrims to Mecca

**DOI:** 10.1093/jtm/taae042

**Published:** 2024-03-08

**Authors:** Saber Yezli, Salleh Ehaideb, Yara Yassin, Badriah Alotaibi, Abderrezak Bouchama

**Affiliations:** Biostatistics, Epidemiology and Scientific Computing Department, King Faisal Specialist Hospital and Research Centre, Riyadh 11564, Saudi Arabia; Experimental Medicine Department, King Abdullah International Medical Research Center, King Saud bin Abdulaziz University of Health Sciences, Ministry of National Guard Health Affairs, Riyadh 11481, Saudi Arabia; Federation of Saudi Chambers Institute, Federation of Saudi Chambers, Riyadh 12711, Saudi Arabia; Ministry of Health, Riyadh 12382, Saudi Arabia; Experimental Medicine Department, King Abdullah International Medical Research Center, King Saud bin Abdulaziz University of Health Sciences, Ministry of National Guard Health Affairs, Riyadh 11481, Saudi Arabia

**Keywords:** Mass gatherings, heat stroke, heat exhaustion, climate change, mortality, incidence, public health, health outcomes, mitigation strategies, ambient temperatures

## Abstract

**Background:**

Global temperatures are on the rise, leading to more frequent and severe heatwaves with associated health risks. Heat-related illnesses (HRIs) are an increasing threat for travellers to hot climate destinations. This study was designed to elucidate the interplay between increasing ambient temperatures, incidence of HRIs and the effectiveness of mitigation strategies during the annual Hajj mass gathering over a 40-year period.

**Methods:**

An observational study was conducted utilizing historical records spanning four decades of meteorological data, and the rates of heat stroke (HS) and heat exhaustion (HE) during the Hajj pilgrimage in Mecca, Saudi Arabia. With an annual population exceeding 2 million participants from over 180 countries, the study analysed temporal variations in weather conditions over two distinct Hajj hot cycles and correlated it with the occurrence of HS and HE. The effectiveness of deployed mitigation measures in alleviating health vulnerabilities between the two cycles was also assessed.

**Results:**

Throughout the study period, average dry and wet bulb temperatures in Mecca escalated by 0.4°C (Mann-Kendall *P* < 0.0001) and 0.2°C (Mann-Kendall *P* = 0.25) per decade, respectively. Both temperatures were strongly correlated with the incidence of HS and HE (*P* < 0.001). Despite the intensifying heat, the mitigation strategies including individual, structural and community measures were associated with a substantial 74.6% reduction in HS cases and a 47.6% decrease in case fatality rate.

**Conclusion:**

The study underscores the escalating climate-related health risks in Mecca over the study period. The mitigation measures’ efficacy in such a globally representative setting emphasizes the findings’ generalizability and the importance of refining public health interventions in the face of rising temperatures.

## Introduction

Rising global temperatures have amplified the frequency and intensity of heatwaves, with significant impacts on human health.^1^^,^^2^ Notably, Europe registered over 70 000 excess deaths in the summer of 2003,^3^ and 61 672 heat-related deaths during the record-breaking summer of 2022.^4^ Current estimations suggest that heat-related excess mortality could approach nearly half a million annually, concentrated largely in Asia and Europe.^5^ In response to these challenges, governmental and non-governmental agencies, such as the World Health Organization and the US Centers for Disease Control and Prevention, have proposed mitigation measures to protect populations from the adverse effects of extreme heat.^6^^,^^7^ Heat-related illnesses (HRIs) are also recognized as an increasing threat for travellers to hot climate destinations, and a number of mitigation measures to protect these travellers have been proposed.^8^

In this global context, the annual Hajj pilgrimage in Mecca, Kingdom of Saudi Arabia (KSA), draws ~2 million pilgrims from over 180 countries, many of whom are unacclimatized to the region’s desert climate. Hajj often aligns with hot cycles in a desert environment, presenting exceptional risks of HRIs.^9^ For instance, in 1987, during a hot cycle, ~1000 heat stroke (HS) deaths occurred within a few days.^10^ In response, KSA introduced a comprehensive range of mitigation measures (Table 1) encompassing individual, structural and community-level interventions. These interventions include promoting hydration, significant infrastructure enhancements and the development of heat-health action plans. However, as climate models project even more severe environmental heat in Mecca,^11^^,^^12^ concerns arise about the sufficiency of current mitigation measures in the face of escalating heat.^13^

**Table 1:** Selected heat illnesses preventive and management measures in Hajj

Intervention	Comment
Sprinklers	Water mist sprays are available throughout the Hajj sites.^13^
Transport	Metro lines have been operational since 2010, transporting pilgrims in the holy sites during the rituals.^31^ Additionally, air-conditioned buses are available for pilgrims during Hajj.^16^
Environmental engineering and building design	Heat mitigation strategies have been integrated into planning and designing/redesigning of pilgrimage sites. These include optimizing buildings layout and open spaces, increasing their capacities and applying reflective coatings to enhance natural ventilation, reduce heat retention, increase shading and reduce crowding. Examples of such interventions include: Expansion of the Grand Mosque notably in the periods 1955–73, 1982–88 and 2008 to date. Redesigning the old Jamaraat Bridge into a new multi-level bridge in 2006, enhancing pedestrians flow management, capacity and shading.^32^ Coating the pedestrian pathway from Mina to Jamarat facilities, encompassing ~3500 square metres in 2019.
Greenery/Tree planting	Since the early 2000s, various initiatives to plant trees and create green spaces in and around the pilgrimage sites have been lunched to provide shade and lower temperatures.^17^
Water and umbrellas	Free water and umbrellas are made available and accessible to all pilgrims.^16^^,^^31^^,^^33^
HRIs management guidelines	The Saudi Ministry of Health has formulated HRI guidelines for healthcare workers (HCWs). These guidelines established in 2009 and updated in 2016 and 2019, encompass pre-hospital management and in-hospital management procedures, enabling proper recognition and handling of HRI cases during the Hajj season.^31^^,^^34^
Education/Risk communication	The Saudi authorities actively elevate awareness among pilgrims and HCWs about HRI risks through educational campaigns pre and during the Hajj season.^16^^,^^31^ These involve distributing brochures to pilgrims upon arrival, establishing trained HCW-operated free phone lines, and advising international agencies to provide pre-arrival awareness programmes for pilgrims’ safety.^31^^,^^33^
Air conditioned heat-resistant tents in Mina and Arafat	Since 1997, the holy site offers temporary accommodation in over 100 000 air-conditioned heat-resistant tents.^35^
Healthcare services	The Saudi government offers free health services during Hajj rituals through 16 hospitals and 128 primary health centres (PHCs), including seven seasonal health facilities. More than 13 000 core HCWs manage these seasonal health facilities, incorporating cooling units for rapid body cooling after exposure to extreme heat.^31^^,^^34^
Health early warning system surveillance	In 2019, the Saudi Ministry of Health introduced the Health Early Warnings System (HEWS) surveillance. HEWS uses both event-based and syndromic surveillance data to rapidly identify potential public health threats, trigger appropriate alerts for prompt epidemiological investigations and monitor the trend of confirmed health issues, including HRI cases.^36^
Updated heatwave emergency plan	Since 2016, the Saudi Ministry of Health has implemented a continually updated heatwave emergency plan.

The present study leverages a robust longitudinal dataset spanning four decades of meteorological and HRIs data in Mecca and comprising an annual population exceeding 2 million individuals. We investigate the temporal variations in weather conditions over two Hajj hot cycles which recur every 20 years and their concomitant impacts on human health. We assess the efficacy of the deployed mitigation measures in alleviating health vulnerabilities associated with extreme heat exposure. By elucidating the relationship between weather conditions, HRIs and the efficacy of mitigation strategies, our research contributes to the expanding knowledge base on health risks posed by extreme heat and HRIs preventive measures.

## Method

### Study site

Mecca is located in a narrow desert valley surrounded by mountains in the Hejaz region of KSA. The city is 70 km from the Red Sea and 277 m above sea level. Mecca is characterized by a hot desert climate, experiencing warm to hot temperatures all year round and fluctuating relative humidity.^14^^,^^15^ Mecca is the site of the Muslim Hajj pilgrimage, which is dictated by the lunar calendar; hence, the event may occur at different seasons across the year. Hajj is performed over 5 days during the 12th month (Dhul-Hijjah) of the Islamic calendar and involves several rituals performed at specific locations (Figure S1). These locations include Mecca city and the holy sites of Mina (≈8 km away), the Plain of Arafat (≈13 km from Mina) and Muzdalifah (≈9 km away from the Plain of Arafat).^16^

### Study design and data sources

This is an observational study of historical records of Mecca meteorological data (1980–2021) and the incidence of HS and HE during Hajj (1980–2019). Daily Dry Bulb (air) Temperature (*T_a_*) (including minimum, mean and maximum) and average daily Wet Bulb Temperature (*T_w_*) in Mecca between 1 January 1985 and 31 December 2021 were extracted from the Saudi National Center for Meteorology database for the Mecca weather station number 41030, located at 39·46 longitudes and 21·26 latitudes. Only average monthly *T_a_* and *T_w_* during Hajj were available for the years 1980–84.^17^*T_a_* refers to the ambient air temperature measures with a standard thermometer, unaffected by moister content. On the other hand, *T_w_* is the temperature registered by a thermometer that has its bulb covered with a wet wick and exposed to moving air. As *T_w_* reflects both temperature and humidity, it is an important indicator of heat stress and the risk of HRIs. This is because in hot and humid conditions the efficiency of evaporative cooling slows and the human body may become unable to maintain a stable core temperature, leading to HRIs.^18^ Aggregate data on the incidence of HS and HE, as well as mortality from HS during Hajj between 1980 and 2019, were extracted from the Saudi Ministry of Health annual reports.^19^ Data on HS and HE during 2020 and 2021 Hajj seasons were excluded as the number of Hajj pilgrims was significantly reduced during these years given the COVID-19 pandemic. When Hajj hot and cold cycles were compared, data from the 1980 and 1981 Hajj season were excluded as these represent only 2 years of a Hajj cold cycle.

### Statistical methods

We summarized the data using descriptive statistics such as means and median for continuous variables and frequencies and percentages for categorical variables. When appropriate, we summarized the data using pooled descriptive statistics. We calculated the estimated pooled proportions using inverse variance weighting approach. Using a normal approximation formula, we calculated the 95% confidence interval (CI) for the pooled estimates. Differences between variables were tested using the Student’s t test, one-way ANOVA, Mann–Whitney U test or the Chi-Square test as appropriate, depending on the variable type and its distribution. Spearman correlation coefficient (r_s_) was used to measure the strength and direction of the relationship between variables. Trends in Mecca’s annual temperatures over the study period were analyzed using the Mann-Kendall trend test. We performed all statistical analyses using R version 3.3.3 software (Foundation for Statistical Computing, Vienna, Austria).

## Results

### Long-term temperature changes in Mecca

#### Monthly and seasonal trends

**Figure 1:**
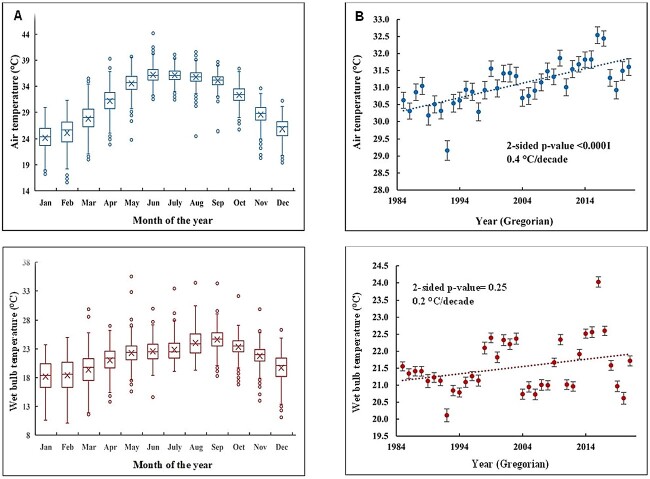
Temperature tends in Mecca (1985–2021). (A) Average monthly air temperatures (*T_a_*) [top] and wet bulb temperatures (*T_w_*) [bottom] in Mecca (1985–2021). Box plots show the median, mean (x), minimum and maximum values, and interquartile range of *T_a_* and *T_w_*. (B) Time series of annual mean air (top) and wet bulb (bottom) temperatures in Mecca (1985–2021) with trendiness, and values represent the average, tow-sided Mann-Kendall *P*-value and decadal trend.

Figure 1A illustrates the variations in average monthly *T_a_* and *T_w_* in Mecca from 1985 to 2021. May through September are the hottest months in Mecca, characterized by a mean monthly *T_a_* exceeding 34.5°C. When Hajj falls within these months, it is considered to be in its ‘hot cycle’, while if the Hajj season falls outside these months, it is considered to be in the ‘cold cycle’. The highest average monthly *T_w_* values are observed in August (mean monthly *T_w_* = 24.0°C ± SD = 2.1), September (mean monthly *T_w_* = 24.6°C ± SD = 1.8) and October (mean monthly *T_w_* = 23.3°C ± SD = 1.6), indicating that these months pose the greatest risk for HS and heat exhaustion (HE).

#### Decadal shifts

An upward trend in both average annual *T_a_* and *T_w_* was observed over time. Each decade saw an increase of 0.4°C in *T_a_* (Mann-Kendall *P* < 0.0001) and 0.2°C in *T_w_* (Mann-Kendall *P* = 0.25), as shown in Figure 1B.

### Temperature trends during the Hajj periods

#### Cycles of heat

**Figure 2:**
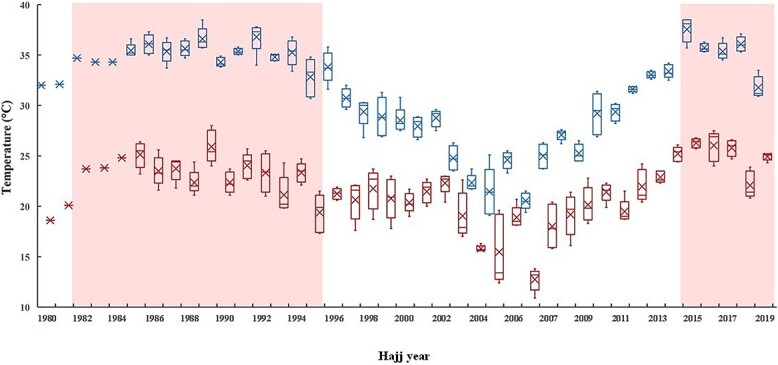
Trend of average daily air (top) and wet bulb (bottom) temperatures during Hajj (1980–2021). Box plots show the median, mean (x), minimum and maximum values, and interquartile range of air temperatures (*T_a_*) and wet bulb temperatures (*T_w_*) during Hajj. Only average monthly *T_a_* and *T_w_* values were available for years 1980–1984. Shaded area represents the Hajj hot cycles [previous hot cycle (1982–1995), current hot cycle (2015–2019)].

Figure 2 presents the trends in average daily *T_a_* and *T_w_* for each Hajj season from 1982 to 2019. This period includes a full previous hot cycle (1982–1995), a complete cold cycle (1996–2014) and a partial current hot cycle (2015–2019). The hot cycles exhibited higher average daily *T_a_* and *T_w_* during Hajj compared with the cold cycle, as detailed in Table 2. Also, during the current hot cycle, average daily *T_w_* during Hajj was higher than in the previous hot cycle (Table 2).

**Table 2:** Average daily air and wet bulb temperatures for Hajj during the hot and cold cycles (1982–2019)

	Mean	SD	*P*-value^a^
*T_a_* (°C)			
Cold cycle (1996–2014)	27.5	3.8	<0.0001^b^
Hot cycle (1982–95 and 2015–19)	35.1	1.6	
*Previous hot cycle (1982–95)*	35.0	1.0	0.67^c^
*Current hot cycle (2015–19)*	35.1	2.1	
*T_w_* (°C)			
Cold cycle (1996–2014)	19.6	2.9	<0.0001^b^
Hot cycle (1982–95 and 2015–19)	23.8	2.0	
*Previous hot cycle (1982–95)*	23.3	1.9	<0.0001^c^
*Current hot cycle (2015–19)*	25.0	1.7	

^a^Student’s *t* test.

^b^Cold cycle vs hot cycle.

^c^Previous hot cycle vs current hot cycle.

*T_a_*: air temperature; *T_w_*: wet bulb temperatures; SD: standard deviation.

#### Extreme heat and dangerous threshold

**Figure 3:**
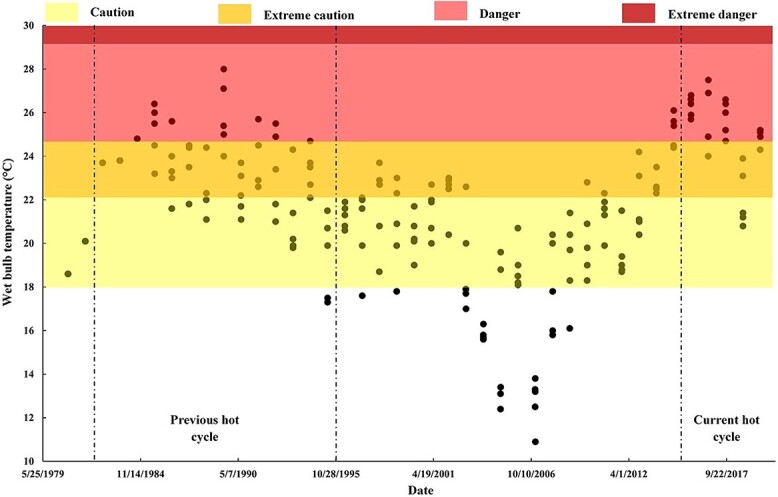
Average daily wet bulb temperature in Hajj and heat stress risk levels during the study period (1980–2019). Average daily wet bulb temperature (*T_w_*) observations during the 5 days of Hajj. Only average *T_w_* values were available for years 1980–84. Bands indicate USNWS heat stress risk level equivalents at 45% relative humidity. Vertical dashed lines indicate bounds of Hajj periods occurring during the hot cycle [previous hot cycle (1982–1995), current hot cycle (2015–2019)].

The highest maximum *T_a_* recorded during the study period was 48.7°C on 13 July 1989, which also had one of the highest daily average *T_a_* (38.5°C) and *T_w_* (27.1°C). The average daily *T_w_* during Hajj surpassed the US National Weather Service (USNWS) danger threshold of 24.6°C on 38 days during the study period, almost half of which occurred in the first five years of the current hot cycle (Figure 3).

### Incidence of HRIs during Hajj

#### Spatial distribution

Most HS and HE cases were concentrated in the holy sites, particularly Mena (Figure S1), which accounted for about two-thirds of all cases (Table S1).

#### Temporal trends

**Figure 4:**
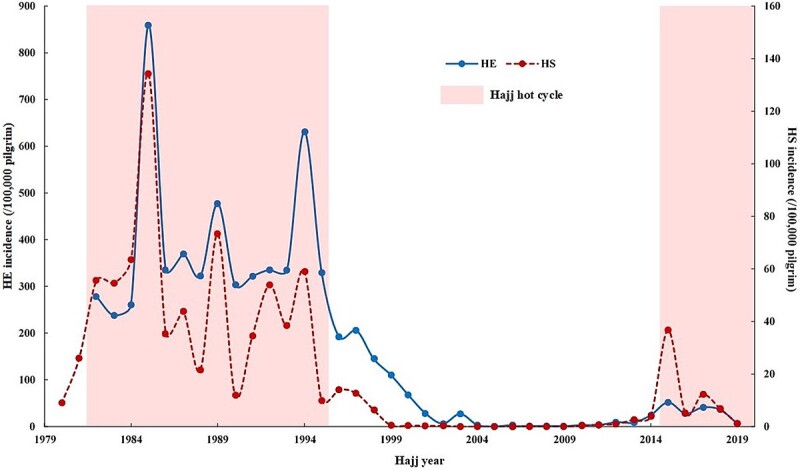
Incidence of heat stroke (HS) and heat exhaustion (HE) during Hajj (1980–2019). Incidence per 100 000 pilgrims of HS [dashed line] and HE [solid line] during Hajj (1980–2019). Shaded area represents the Hajj hot cycles [previous hot cycle (1982–1995), current hot cycle (2015–2019)]. Incidence of HE for the years 1980 and 1981 were not available.

Rates of HS and HE generally increased in warmer cycles with a strong positive correlation between the incidence rates of these two conditions (Spearman’s rho: rs = 0.9, *P* < 0.001). In the previous hot cycle, HS and HE rates peaked at 134.2 and 858.8 per 100 000 pilgrims, respectively, in August 1985 (Figure 4), under an average daily *T_w_* of 25.1°C. In contrast, the cold cycle (1996–2014) saw a substantial drop in HS and HE rates (Table 3). However, rates began to rise again as Hajj entered another hot cycle in 2015. The current hot cycle has shown fewer incidences of HS and HE than the previous hot cycle but more than the cold cycle (Table 3).

**Table 3:** Incidence of heat stroke (HS) and heat exhaustion (HE) per 100 000 pilgrims in Hajj during the hot and cold cycles (1982–2019)

	Mean	SD	Median	IQR	*P*-value^a^
HS					
Cold cycle (1996–2014)	2.1	4.2	0.32	2.2	<0.0001^b^
Hot cycle (1982–95 and 2015–19)	39.6	31.8	36.7	43.6	
*Previous hot cycle (1982–95)*	49.3	30.9	48.9	28.9	0.007^c^
*Current hot cycle (2015–19)*	12.5	14.1	6.9	21.3	
HE					
Cold cycle (1996–2014)	42.1	66.5	7.4	56.3	<0.0001^b^
Hot cycle (1982–95 and 2015–19)	292.5	214.3	321.9	283.6	
*Previous hot cycle (1982–95)*	385.4	168.1	331.9	99.2	<0.0001^c^
*Current hot cycle (2015–19)*	32.7	17.1	36.3	29.2	

^a^Mann-Whitney U test.

^b^Cold cycle vs hot cycle.

^c^Previous hot cycle vs current hot cycle.

SD: standard deviation; IQR: interquartile range.

### Correlation between ambient temperature and incidence of HRIs during Hajj

A strong overall correlation exists between the incidence of HS and HE and average daily *T_a_* and *T_w_* during Hajj (Table 4). However, this correlation varies depending on the cycle in which Hajj occurs.

**Table 4:** Correlation between the incidence of heat stroke (HS) and heat exhaustion (HE) and average daily air and wet bulb temperatures during Hajj

	Previous hot cycle (1980–95)		Cold cycle (1996–2014)		Current hot cycle (2015–19)		All (1980–2019)	
	r_s_	*P*-value	r_s_	*P*-value	r_s_	*P*-value	r_s_	*P*-value
HE								
Average daily *T_a_*	0.61	0.021	0.66	0.002	0.70	0.188	0.76	>0.0001
Average daily *T_w_*	0.10	0.725	0.57	0.008	0.50	0.391	0.54	>0.0001
HS								
Average daily *T_a_*	0.27	0.349	0.93	>00001	0.70	0.188	0.86	>0.0001
Average daily *T_w_*	0.74	0.002	0.74	>0.0001	0.50	0.391	0.73	>0.0001

Spearman’s rho: r_s_

*T_a_*: air temperature; *T_w_*: wet bulb temperatures.

### Mortality rates due to HS during Hajj

**Figure 5:**
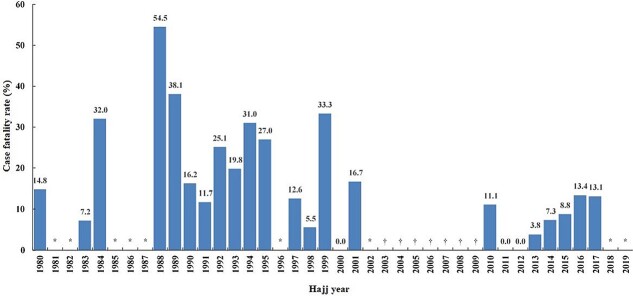
Case fatality rates (CFR) for heat stroke (HS) among pilgrims during Hajj (1080–2019). Bar chart representing CFR of HS among pilgrims during Hajj. *: Not reported; †: No HS cases during the pilgrimage.

Data from the Saudi Ministry of Health reveal varying Case Fatality Rates (CFRs) in relation to HS during the Hajj pilgrimage (Figure 5), particularly during different temperature cycles. Over the past four decades, the overall CFR for HS during Hajj stood at nearly 5% (pooled CFR =4.9%, 95% CI =3.8–6.1). CFR peaked during the previous hot cycle (pooled CFR =19.1%, 95% CI =18.2–20.0), with notable spikes in 1985 and 1988. During the 1985 Hajj, nearly half of all HS cases (47.5%) died.^10^ For the 1988 Hajj, some documented a staggering CFR of 54.4%,^20^ although other sources suggest lower rates.^17^ The CFRs were lower during the subsequent cold cycle, with a pooled rate of 2.8% (95% CI = 0.5–5.1). Rates increased in the current hot cycle (pooled CFR =10.0% (95% CI =8.2–11.7), but remain substantially lower than the previous hot cycle.

### Sensitivity analysis: comparing early years of hot cycles

To account for variations in the duration of different hot cycles, a sensitivity analysis was performed. This focused on the first 5 years of the previous hot cycle (1982–1986) and the corresponding initial 5 years of the current hot cycle (2015–2019), specifically when Hajj took place in August and September. The sensitivity analysis essentially corroborated the principal results (Tables S2–4), as summarized below:

#### Temperature trends

Increase was observed in the mean *T_w_* during the first 5 years of the current hot cycle compared with the previous hot cycle (Table S2).

#### Incidence rates of HS and HE

The rates of both HS and HE were lower in the current hot cycle compared with the previous one (Table S3).

#### Correlation with ambient temperature

In the previous hot cycle, strong positive correlations were found between *T_a_* and *T_w_*, and the incidences of HE and HS, respectively (Table S4). No such correlations were found during the current hot cycle, highlighting a disconnect between temperature and the incidence of HRIs in the more recent years.

#### Implications and conclusions

The sensitivity analysis reinforces the main findings and suggests that while the current hot cycle is associated with higher mean *T_a_* and *T_w_*, it has fewer incidences of HS and HE. Furthermore, the absence of strong correlations between temperature and incidence rates in the current hot cycle suggests more effective heat management strategies.

## Discussion

This study investigated how escalating environmental heat affects HRIs among Hajj pilgrims in Mecca. We evaluated the climate and health data for ~40 years to understand these links and the efficacy of heat mitigation strategies.

Our findings reveal an upward trajectory in the average annual *T_a_* and *T_w_* of Mecca over the study period. Specifically, each decade saw increases of 0.4°C in *T_a_* and 0.2°C in *T_w_*, consistent with earlier Mecca temperature trend studies.^11^^,^^21^ For instance, Kang et al.^11^ identified a 2°C rise in Mecca in both *T_a_* and *T_w_* over 30 years (1984–2013). This exceptional warming surpasses global averages and is primarily attributed to increased anthropogenic greenhouse gas emissions.^11^ Predictions under future climate scenarios further emphasize the escalating heat risk, with *T_w_* reaching potentially lethal levels for humans.^11^^,^^22^ Under business-as-usual scenarios, Mecca could experience *T_w_* of up to 32°C and *T_a_* exceeding 55°C.^22^^,^^23^ These projections highlight an imminent health risk to unacclimatized travellers to the Hajj pilgrimage and have broader global implications.^8^^,^^11^^,^^12^^,^^22^

During Hajj, the average daily *T_a_* and *T_w_* were markedly higher in hot cycles compared with cold cycles, with record-breaking values observed in the current hot cycle. Notably, half of the Hajj days which exceeded the USNWS 24.6°C danger threshold were during the initial 5 years of the current hot cycle. This alarming trend, mirroring global heat patterns due to climate change,^22^^,^^24^ exacerbates the risk of HRIs and associated mortality among already vulnerable Hajj pilgrims.^9^^,^^16^ Recent research has indicated that heat-attributed mortality among pilgrims is 4.5 times higher than among Mecca’s resident population.^9^ This heightened vulnerability can be attributed to the pilgrims’ lack of acclimatization to Mecca’s climate and to specific risk factors associated with the Hajj rituals.^16^ As temperatures continue to escalate and predictions suggest further worsening, it becomes increasingly crucial to understand the compounding effects of intensifying heat on the health of these vulnerable pilgrims.

Our analysis demonstrates a robust positive correlation between HS and HE incidence and the average daily *T_a_* and *T_w_* during Hajj, notably in cold and previous hot cycles. This underscores the direct link between rising temperatures and an escalating risk of HRIs.^25^ The previous hot cycle showed peak incidence rates of HS and HE, which declined in the following cold cycle but have risen again in the current hot cycle. HRIs are expected during Hajj even during the cold cycle given pilgrims’ susceptibilities^16^ and the desert climate of Mecca where temperatures remain relatively elevated most of the year. This is practically true in the periods when Hajj has just left, or about to enter, a hot cycle, where temperatures remain high. The overall CFR for HS was 5%, aligning closely with a recent systematic review of HS among Hajj pilgrims,^26^ but lower than in other settings.^27^ This difference may be partially attributed to quick access to healthcare during Hajj. CFR for HS was highest in the previous Hajj hot cycle (19.1%) and lowest during the cold cycle (2.8%).

Interestingly, despite climbing temperatures, we observed lower incidence rates of HE and HS, as well as reduced CFRs in the current hot cycle compared with the previous one. This suggests that mitigation measures implemented during the cold cycle have been effective in curbing HRIs and fatalities. Population-level strategies like heat action plans and education campaigns have successfully prevented HRIs and reduced mortality across diverse economic settings.^28–30^ For instance, the heat action plan introduced in France resulted in nearly 4400 fewer heat-related deaths than expected during the 2006 heatwave, based on historical data.^29^ However, it is crucial to acknowledge that despite mitigation efforts, the incidence rates and CFRs for HRIs in the recent hot cycle remain higher than those in the cold cycle. This suggests that worsening heat may be outpacing the comprehensive mitigation strategies in place. Given the projections, this is especially crucial for Hajj. Even under the Paris Agreement target of limiting global warming to 1.5°C, the risk of HS among pilgrims at higher *T_w_* is projected to multiply 5-fold, and 10-fold in a 2°C warmer world.^12^

Our study has certain limitations that merit consideration. We relied on historical and aggregate data, which may not capture individual-level variations and specific risk factors. Moreover, our focus on HS and HE as primary health indicators could potentially neglect the role of other pre-existing chronic medical conditions aggravated by heat, as well as milder forms of HRIs. Furthermore, attributing the observed impact to a particular mitigation measure was beyond the scope of this study. Future research could adopt a multifaceted approach, incorporating a broader range of health indicators and exploring their links with specific risk factors and mitigation measures.

Despite its limitations, this study has significant public health implications. It adds to the accumulating evidence highlighting the escalating severity of environmental heat and the effectiveness of mitigation strategies in reducing the incidence and severity of HRIs. Intriguingly, our data suggest that the intensifying heat may be outpacing current mitigation efforts, signalling a need to recalibrate existing approaches. The Hajj pilgrimage serves as a unique microcosm for studying heat-related risks, as it attracts millions of pilgrims from over 180 countries. This international diversity means that our findings are not only locally relevant but can also be generalized to a global population. Therefore, these insights hold immense value for the planning and refinement of public health interventions worldwide. In addition, our work highlights the risk of HRIs for travellers to attend mass gatherings, or for other purposes, in hot climates and the importance of physicians discussing these illnesses with such travellers and the strategies to prevent or minimize their impact. This may include advice on staying hydrated in such settings, staying indoors during the hottest times of the day, seeking shaded areas whenever possible and cooling down in air-conditioned locations. In additions, travel physicians could also raise awareness among their clients on how to recognize and manage the symptoms of HRIs early before the conditions worsen.

## Supplementary Material

Supplementary_data_taae042

## Data Availability

Data will be available from the corresponding author on a reasonable request and on an appropriate data sharing agreement, subject to approval by the Saudi National Center for Meteorology.
