# Supplementary material for: Escalating climate-related health risks for Hajj pilgrims to Mecca
Source: J Travel Med. 2024 Mar 8;31(4):taae042. doi: 10.1093/jtm/taae042 (PMC11149718; doi:10.1093/jtm/taae042)
Supplement: Supplementary_data_taae042 [file supplementary_data_taae042.docx]

**The Interplay of Rising Temperatures, Health Outcomes, and Mitigation Efficacy: A 40-year Analysis from the Hajj**

**Table S1. Location of heat stroke and heat exhaustion cases during Hajj**

| **Hajj sites** | **Heat stroke** | | **Heat exhaustion** | |
| --- | --- | --- | --- | --- |
|  | **EPP** | **95% CI** | **EPP** | **95% CI** |
| **Holy sites** | 85.3% | 83.3-87.1 | 99.2% | 98.2-99.4 |
| Mina | 62.0% | 59.8-64.8 | 68.1% | 66.6-69.5 |
| Arafat and Muzdalifa | 18.2% | 16.2-20.2 | 28.6% | 27.3-30.0 |
| **Mecca city** | 14.3% | 12.5-16.1 | 0.8% | 0.5-1.0 |
| **Medina city** | 0.1% | 0.0-0.3 | 0.1% | 0.03-0.2 |

EPP: estimated pooled proportions, CI: confidence interval

**Sensitivity analysis**

**Table S2. The average daily air and wet bulb temperatures for Hajj during the previous 5-years of the previous and current Hajj hot cycles**

|  | **Mean** | **SD** | **p-value^a^** |
| --- | --- | --- | --- |
| ***T_a_* (°C)** |  |  |  |
| *Previous hot cycle (First 5 years 1982-1986)* | 34.9 | 0.8 | 0.97 |
| *Current hot cycle (First 5 years 2015-2019)* | 35.0 | 2.1 |  |
| ***T_w_* (°C)** |  |  |  |
| *Previous hot cycle (First 5 years 1982-1995)* | 24.2 | 1.0 | **0.02** |
| *Current hot cycle (First 5 years 2015-2019)* | 25.0 | 1.7 |  |

^a^Student's t test;

*T_a_*: air temperature; *T_w_* wet bulb temperatures; SD: standard deviation

**Table S3. The incidence of heat stroke and heat exhaustion per 100,000 pilgrims in Hajj during the previous 5-years of the previous and current Hajj hot cycles**

|  | **Mean** | **SD** | **Median** | **IQR** | **p-value^a^** |
| --- | --- | --- | --- | --- | --- |
| **Heat stroke** |  |  |  |  |  |
| *Previous hot cycle (First 5 years 1982-1986)* | 68.7 | 38.1 | 55.6 | 53.9 | **0.008** |
| *Current hot cycle (First 5 years 2015-2019)* | 12.5 | 14.1 | 6.9 | 21.3 |  |
| **Heat exhaustion** |  |  |  |  |  |
| *Previous hot cycle (First 5 years 1982-1986)* | 394.2 | 262.2 | 278.1 | 347.9 | **0.008** |
| *Current hot cycle (First 5 years 2015-2019)* | 32.7 | 17.1 | 36.3 | 29.2 |  |

^a^Mann-Whitney U test;

SD: standard deviation; IQR: interquartile range

**Table S4. Correlation between the incidence of heat stroke and heat exhaustion and average daily air and wet bulb temperatures in Hajj the previous 5-years of the previous and current Hajj hot cycles**

|  | **Previous hot cycle (First 5 years 1982-1986)** | | **Current hot cycle (First 5 years 2015-2019)** | |
| --- | --- | --- | --- | --- |
|  | **r_s_** | **p-value** | **r_s_** | **p-value** |
| **Heat Exhaustion** |  |  |  |  |
| Average daily *T_a_* | 0.87 | **0.045** | 0.70 | 0.188 |
| Average daily *T_w_* | 0.10 | 0.873 | 0.50 | 0.391 |
| **Heat Stroke** |  |  |  |  |
| Average daily *T_a_* | -0.27 | 0.741 | 0.70 | 0.188 |
| Average daily *T_w_* | 0.90 | **0.003** | 0.50 | 0.391 |

Spearman’s rho: r_s_

*T_a_*: air temperature; *T_w_* wet bulb temperatures;

**Fig S1**

**Fig S1. The Hajj journey**


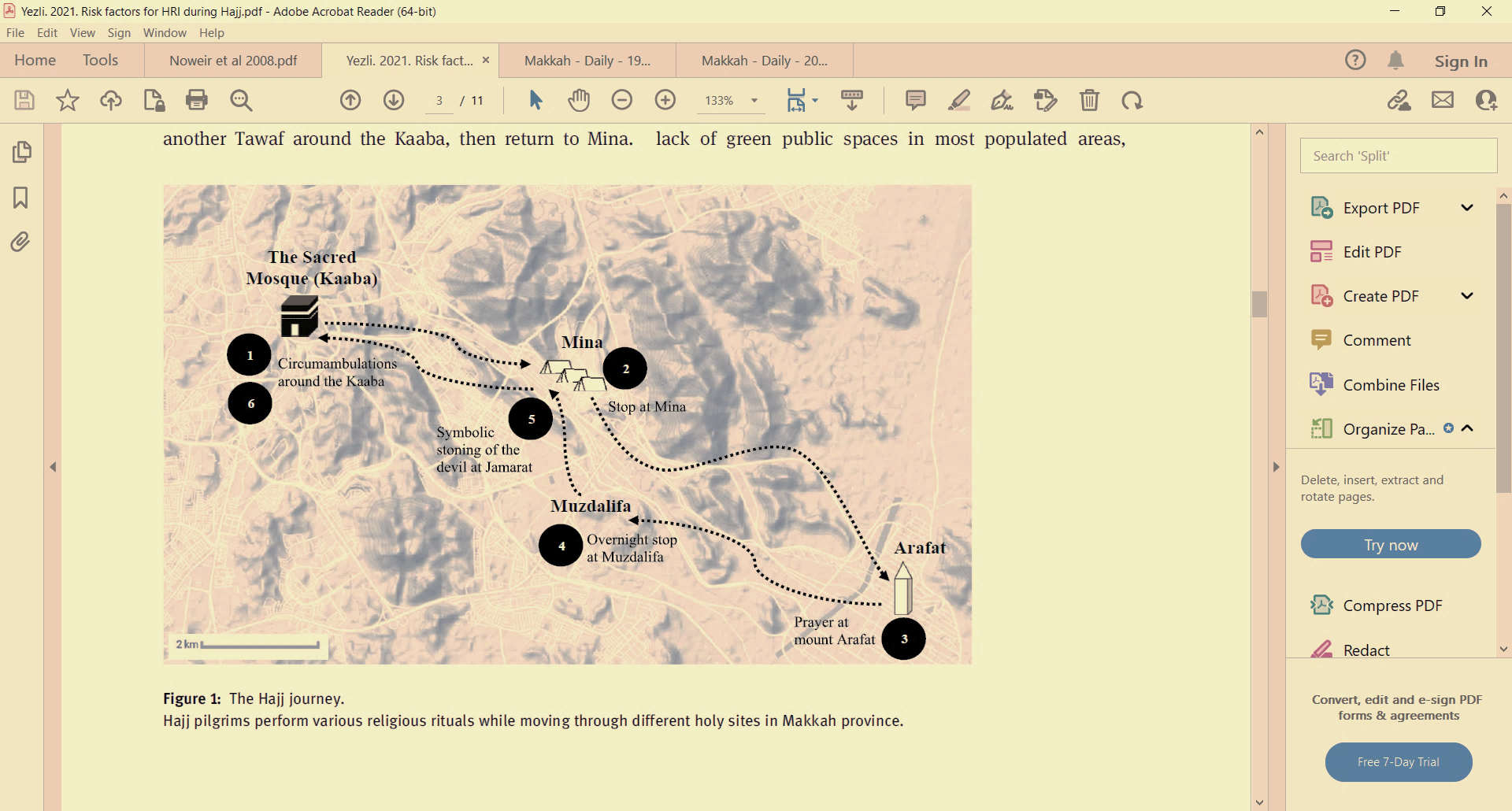


Pilgrims perform various religious rites while moving from the Sacred Mosque in Mecca city and throughout the holy sites of Mina, Arafat and Muzdalifa.^1^

**References**

1. Yezli S. Risk factors for heat-related illnesses during the Hajj mass gathering: an expert review. *Rev Environ Health* 2021.
